# Knockdown of Pentraxin 3 suppresses tumorigenicity and metastasis of human cervical cancer cells

**DOI:** 10.1038/srep29385

**Published:** 2016-07-05

**Authors:** Tsung-Ho Ying, Chien-Hsing Lee, Hui-Ling Chiou, Shun-Fa Yang, Chu-Liang Lin, Chia-Hung Hung, Jen-Pi Tsai, Yi-Hsien Hsieh

**Affiliations:** 1 Department of Obstetrics and Gynecology, Chung Shan Medical University Hospital, Taichung, Taiwan; 2 Department of Obstetrics and Gynecology, School of Medicine, College of Medicine, Chung Shan Medical University, Taichung, Taiwan; 3 School of Chinese Medicine, College of Chinese Medicine, China Medical University, Taichung, Taiwan; 4 Division of Pediatric Surgery, Department of Surgery, Children’s Hospital of China Medical University, Taichung, Taiwan; 5 School of Medical Laboratory and Biotechnology, Chung Shan Medical University, Taichung, Taiwan; 6 Institute of Medicine, Chung Shan Medical University, Taichung, Taiwan; 7 Institute of Biochemistry, Microbiology and Immunology, Chung Shan Medical University, Taichung, Taiwan; 8 School of Medicine, Tzu Chi University, Hualien, Taiwan; 9 Division of Nephrology, Department of Internal Medicine, Dalin Tzu Chi Hospital, Buddhist Tzu Chi Medical Foundation, Chiayi, Taiwan; 10 Department of Biochemistry, School of Medicine, Chung Shan Medical University, Taichung, Taiwan; 11 Clinical laboratory, Chung Shan Medical University Hospital, Taichung, Taiwan

## Abstract

Pentraxin 3 (PTX3) as an inflammatory molecule has been shown to be involved in immune response, inflammation, and cancer. However, the effects of PTX3 on the biological features of cervical cancer cells *in vitro* and *in vivo* have not been delineated. Immunohistochemical staining showed that increased PTX3 expression was significantly associated with tumor grade (P < 0.011) and differentiation (P < 0.019). Knocking down PTX3 with lentivirus-mediated small hairpin RNA (shRNA) in cervical cancer cell lines resulted in inhibited cell viability, diminished colony-forming ability, and induced cell cycle arrest at the G2/M phase of the cell cycle, along with downregulated expression of cyclin B1, cdc2, and cdc25c, and upregulated expression of p-cdc2, p-cdc25c, p21, and p27. Furthermore, knockdown of PTX3 significantly decreased the potential of migration and invasion of cervical cancer cells by inhibiting matrix metalloproteidase-2 (MMP-2), MMP-9, and urokinase plasminogen activator (uPA). Moreover, *in vivo* functional stud*i*es showed PTX3-knockdown in mice suppressed tumorigenicity and lung metastatic potential. Conversely, overexpression of PTX3 enhanced proliferation and invasion both *in vitro* and *in vivo*. Our results demonstrated that PTX3 contributes to tumorigenesis and metastasis of human cervical cancer cells. Further studies are warranted to demonstrate PTX3 as a novel therapeutic biomarker for human cervical cancer.

Cervical cancer is a common cancer in women and is a major cause of mortality worldwide^1^. Moreover, cervical cancer is the seventh most common cause of death from cancer in women and the tenth most common cause of death overall according to the analysis of the Taiwan Ministry of Health and Welfare in 2013. Reports have suggested that certain types of human papillomavirus (HPV) such as HPV 16 and 18 are strongly associated with invasive squamous cells carcinoma and have the ability to transform normal cervical cells into neoplastic cells. However, the viral infection does not sufficiently explain cancer progression^2^. The prognosis of patients with cervical cancer is largely dependent on the stage of the cancer. The cancer stage at diagnosis determines treatment options and has a strong influence on the length of survival^2^. Understanding the molecular basis of cervical cancer should significantly improve the diagnosis and management of these cervical tumors and eventually lead to the development of more specific and effective treatment modalities^3^.

This study focused on the biological function of PTX3 in proliferative and invasive human cervical cancer. Pentraxin consists of a set of soluble proteins and represents the prototypic components of humoral innate immunity and are divided into short pentraxin and long pentraxin groups based on the primary structure of the promoter^4^. The long PTX3 is produced by innate immunity cells that interacts with several ligands and plays an essential role in innate immunity, tuning inflammation, and matrix deposition^5^. PTX3 has been suggested to play an important role in tumor-associated inflammation. Moreover, PTX3 expression has been identified as a new diagnostic and prognostic biomarker of various types of cancers, including glioma^6^, prostate^7^, lung^8^, soft tissue liposarcoma^9^, and pancreatic cancer^10^. Recent evidence shows that knockdown of PTX3 results in inhibition of proliferation and invasion of lung cancer cells through suppression of the protein kinase B, also known as AKT and NF-ĸB signaling pathways^11^. Additionally, the clinical and biological function of PTX3 showed that it has the potential to be a prognostic biomarker with a tumor promoting activity factor in bone metastatic breast cancer.

For the cervical cancer cells to metastasize at as distant location, the malignant cells must have the ability to migrate and invade, destroy intercellular relationships, lyse extracellular matrix (ECM), and facilitate migration of endothelial cells and capillary lumen formation^12^,^13^. Matrix metallopeptidase-9 (MMP-9) MMP-2, and urokinase plasminogen activator (μPA) have been reported to play a key role in degrading the ECM, by allowing metastatic cells to access the vasculature, invade and migrate into the target organ, and thereby result in tumor metastasis of cervical cancers^14^,^15^. Recently, evidence suggested that positive expression of MMP-9 was associated with PTX3 expression in lung adenocarcinoma^11^, which indicates that PTX3 could be positively associated with tumor grade and severity of malignancies, and modulating inflammation. PTX3 increased the migratory capacity of breast cancer cells^16^. PTX3 can also regulate head and neck cancer cell metastasis by modulating the expression of fibronectin and MMP-9, in which contributes to NF-κB-promoted tumor metastasis^17^. However, the role of PTX3 in cervical cancer and the mechanism responsible for the oncogenic roles of PTX3 remain unknown. In this study, we further investigate the clinical significance and biological function of PTX3 in cervical cancer cells *in vitro* and *in vivo*.

## Materials and Methods

Antibodies and reagents. Anti-p-cdc25c (sc-12354; 1:1000), anti-p21 (sc-397; 1:1000), anti-p27 (sc-528; 1:1000), anti-MMP-2 (sc-53630; 1:1000), anti-MMP-9 (sc-6840; 1:1000), anti-uPA (sc-14019; 1:1000) and anti-β-actin (sc-47778; 1:2000) antibodies were Santa Cruz Biochemicals (Santa Cruz, CA). Anti-cyclin B1 (05-373SP; 1:1000), Anti-cdc25C (05-507SP; 1:1000), anti-cdc2 (06-923SP; 1:1000) antibodies were purchased from Merck Millipore Corporation (Darmstadt, Germany). Anti-PTX3 (AF1826; 1:1000) antibodies and human recombinant-PTX3 protein (1826-TS) were purchased from R&D Technology (Beverly, MA).

### Cell culture

C33A, HeLa, and SiHa cervical cancer cell lines were purchase from Bioresources Collection and Research Center, Food Industry Research and Development Institute (Hsinchu, Taiwan). HeLa cells was growth in DMEM/F12 mediums, SiHa cells was growth in DMEM mediums and C33A cells was growth in RPMI mediums, supplemented with 10% fetal bovine serum (Gibco), 100 units/mL penicillin, 100 μg/mL streptomycin and maintained at 37 °C with 5% CO_2_.

### Human cervical cancer tissue array and immunohistochemistry

Anti-PTX3 antibody was used for immunohistochemical (IHC) detection of the expression of PTX3 protein in human cervical tissue microarrays (CR804; US Biomax, Inc) and analyzed according to the manufacturer’s suggested protocols. Cervical tissues were subjected to heat induced epitope retrieval with Basic Antigen Retrieval Reagent (R&D Technology, Beverly, MA), stained for immunosignals with DAB kit (R&D Technology, Beverly, MA), and counterstained with Gill’s hematoxylin. The PTX3 expression was calculated from the mean of the two independently conducted assessments.

### shRNA clones and lentivirus infection

shRNA-Luc and shRNA-PTX3 clones (TRCN0000149744) were obtained from the National RNAi Core Facility (Institute of Molecular Biology/Genomic Research Center, Academia Sinica, Taipei, Taiwan). Briefly, 2 × 10^5^ cells were seeded in 6 cm dish overnight. Before lentivirus infection, the culture media was changed to fresh media containing 8 μg/mL of polybrene. After the fresh media change, infected cells were incubated overnight and infected cells were selected with 2 μg/mL of puromycin for 5 days. After selection, western blotting was performed to confirm if the knockdown was effective.

### Plasmids DNA and transfection

A human pENTER-PTX3 clone was purchased ViGene Bioscience (Rockville, USA). For plasmid transfection, at 80% confluent cells were transfected with pENTER vector (Neo) or pENTER–PTX3 vector (PTX3) using Jet-PEI reagent according to manufacturer’s instructions. The transfected cells were cultured in complete medium for 16 h and then, in normal growth medium for additional 48 h before performing functional assays or harvesting cell lysates for protein expression analyses.

### Cell viability and clonogenic assay

Cells (2 × 10^4^ cells per well) were seeded in each well of a 24-well plate. After a 24 h incubation, cells were incubated with MTT reagent (0.5 mg/mL) at 37 °C for 2 h. Cells were transferred to a 96-well microplate reader (Bio-Rad Laboratories, Inc., Hercules, CA) and the numbers of live and dead cells were recorded at 24, 48, and 72 h. The experiment was performed in triplicate. To determine long-term effects, cells were seeded into 6-well dishes (1 × 10^3^ cells per well) and cultured for 2 weeks. Colonies formed in each well were fixed with methanol for 15 mins, which were then stained with 0.05% Giemsa for 1 hour. Each cell group was cultured in triplicate and surviving colonies (>50 cells/colony) were scored visually and counted.

### Flow cytometry

For cell cycle analysis, SiHa and HeLa cells were plated in 6 cm culture dish (4 × 10^5^ cells). After 24 h incubation, adherent cells were harvested and fixed in 70% ethanol at 4 °C overnight. Cells were incubated with RNase A at 37 °C for 20 mins, and then stained with propidium iodide (PI). The cells were then measured by flow cytometry using FACScan (BD Biosciences, San Jose, CA, USA) and were analyzed using the CellQuest software (Becton Dickinson, Franklin Lakes, NJ, USA). The experiments were independently performed three times.

### *In vitro* migration and invasion assay

An *in vitro* migration and invasion assay was performed using a 48-well Boyden chamber as previously described^18^. For knockdown PTX3 assay, approximately 5 × 10^5^ SiHa and HeLa cells were added to the upper chamber in serum free media. The lower compartment was filled with serum-free media containing 10% FBS. For recombinant PTX3 and transfection PTX3 assay, approximately 1 × 10^5^ HeLa cells were added to the upper chamber in serum free media containing 100 μg/ml Rh-PTX3. The lower compartment was filled with serum-free media containing 10% FBS. The assays were performed with or without Matrigel (BD Biosciences, San Jose, CA, USA), respectively. All cells were seeded in the upper part of the Boyden chamber and incubated for 12 h for migration and 24 h for invasion. These cells were fixed with 100% methanol and stained with 0.05% Giemsa for 30 mins. The migratory phenotypes were determined by counting the cells that migrated to the lower side of the filter by using microscopy at x400. Thirteen fields were counted for each filter and each sample was assayed in triplicate.

### Semi-quantitative reverse transcription-polymerase chain reaction (RT-PCR) analysis

Total RNA was extracted from shLuc and shPTX3 stable cells using the TRIzol™ reagent (Invitrogen, Carlsbad, CA). Complementary DNA was synthesized from 2 μg of total RNA using the SuperScript III Reverse Transcriptase (Invitrogen). Human PTX3 mRNA (Gene number: NM_002852) was amplified using the sense primer 5′-CTGTATCTCAGCTACCAATCCA-3′ and the antisense primer 5′-TTGCTAAGAACACTATCCCAGA-3′. The polymerase chain reaction (PCR) was carried out as follows: 32 cycles of 95 °C for 30 seconds, 54 °C for 30 seconds, and 72 °C for 1 mins, followed by a 10 mins extension stage at 72 °C. PCR products were electrophoresed through agarose gels and analyzed by computerized densitometry scanning of the images using the Quantity-One imaging software normalized with internal β-actin.

### Western blotting

Total protein was isolated from knockdown PTX3 SiHa/HeLa cells for 5 days, recombinant-PTX3 (Rh-PTX3) and overexpression PTX3 treated HeLa cells for 48 h using NETN buffer (150 mM NaCl, 1% NP-40, and 50 mM Tris [pH 7.4]) containing 1 mM β-glycerophosphate, 2.5 mM sodium pyrophosphate, 1 mM NaF, 1 mM Na_3_VO_4_, and protease inhibitor cocktail. Protein levels were quantified using Bradford assay reagent according to the manufacturer’s instructions. Cell lysates in SDS-NETN buffer were subjected to 10% or 12% SDS-PAGE analysis and electrophoretically transferred to polyvinylidene difluoride membranes. The membranes were blocked with 5% non-fat milk and incubated with antibodies. Signals were detected via enhanced chemiluminescence by using Immobilon Western-HRP Substrate (Millipore, Billerica, USA). Relative band intensities were determined by quantitation of each band with a Luminescent Image Analyzer LAS-4000 mini.

### *In vivo* tumorigenicity assay

Four-week-old female BALB/c nude mice were purchased from the National Laboratory Animal Center (Tainan, Taiwan). All animal studies were conducted according to the protocols approved by the Institutional Animal Care and Use Committee (IACUC) of Chung Shan Medical University. Prior to injection, 10 nude mice were randomised to two groups: shLuc-SiHa cells group (n = 5) and shPTX3-SiHa cells group (n = 5). A total of 5 × 10^6^ shLuc- or shPTX3-SiHa cells in 0.1 mL of saline were subcutaneously injected into the left flank of the nude mice. To assess the efficacy of Rh-PTX3 on tumorigenicity. Three days following control and Rh-PTX3 treated SiHa cell inoculation, the mice began to receive daily i.p. injection with 50 μg of Rh-PTX3 (0.05 ml saline) in 3 days a week for 16 days, and same volume of saline was given as control. Tumor size was measured using a digital vernier caliper. Tumor volume was calculated according to the following formula: mm^3^ = d^2^ × L/2, where d and L represent the shortest and longest diameters, respectively. The mice were sacrificed after 16 or 28 days and the tumors were removed. Tumor tissue sections were prepared, and immunoreactivity was analyzed as above using Ki67 staining.

### *In vivo* lung metastasis assay

Six-week old female severe combined immunodeficiency (SCID) mice were purchased from National Laboratory Animal Center (Tainan, Taiwan). The shLuc-SiHa cells (n = 5) and shPTX3-SiHa cells (n = 5) were injected into tail veins of SCID mice at the density of 1 × 10^6^ in 0.1 ml saline for each cell line. To assess the efficacy of Rh-PTX3 on tumor metastasis, 1 × 10^6^ control and Rh-PTX3 treated SiHa cells were injected into SCID mice (n = 5) via tail vein to imitate tumor metastasis. Six days before tumor cell inoculation, the mice began to receive daily intraperitoneal injection with 50 μg of Rh-PTX3 (0.05 ml saline) in 3 days a week for 35 days, and the same volume of saline was given as control. Mice were killed after 35 and 63 days and lung tissues were resected and photographed. To visual the cancer cells metastasis, lung tissues were harvested, embedded with paraffin, fixed in formalin, and processed for H&E staining. Lung metastases were counted on one H&E stained lung section from each mouse.

### Statistical analysis

Quantitative data were expressed as mean ± SEM. Analysis of variance (ANOVA) with Student’s t-test was used to determine the significant differences among experimental groups, *P < 0.05 or **P < 0.01 was considered significant

## Results

### Expression of PTX3 in human cervical cancer cells and tissues

**Figure 1:**
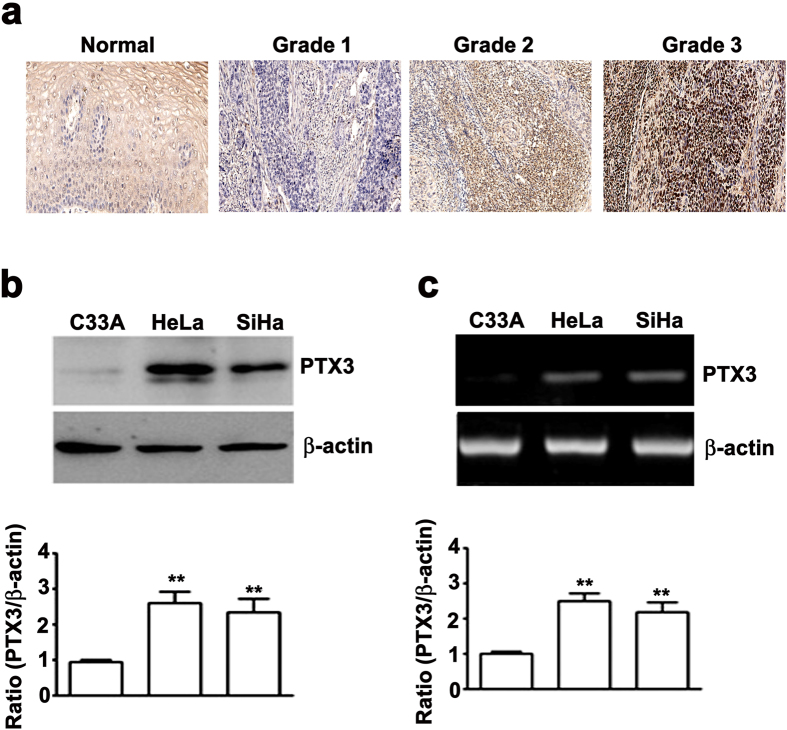
PTX3 is highly expressed in human cervical cancer tissues and cell lines. (**a**) Representative PTX3 expression stained mounted section containing different grades of cervical cancer tissues (Grade I, II, III) and normal cervical tissues by immunohistochemistry staining (x40) (**b**) Total lysate from C33A, HeLa, and SiHa analyzed by western blotting to detect PTX3 expression. (**c**) Semi-quantitative RT-PCR assay. β-actin was used as internal control for equal loading. Values are expressed as the mean ± SE of three independent experiments. **P < 0.01.

PTX3 expression was higher in cervical cancer tissues compared with normal cervical tissues (Fig. 1a). In addition, PTX3 was strongly expressed in grade 2 or grade 3 cervical cancer tissues on tissue microarray (TMA); however, PTX3 was weakly expressed in grade 1 cervical cancer tissues (Fig. 1a). Furthermore, the association between PTX3 expression and clinicpathological parameters was analyzed in 60 cervical cancer patients (Table 1). Statistical analyses suggested that PTX3 expression strongly correlated with tumor grade (P < 0.011) and tumor differentiation (P < 0.019), whereas the expression of PTX3 was not significantly different between age groups. To understand of PTX3 is associated with cervical cancer progression. Western blotting analysis showed a significant increased expression of PTX3 protein in SiHa and HeLa cells compared with C33A cells (Fig. 1b). Using RT-PCR assay, the mRNA levels were higher in SiHa and HeLa cells compared to C33A cells (Fig. 1c). The expression levels of endogenous PTX3 protein and mRNA were positively correlated with cell proliferation, migration and invasion (Supplementary Fig. 1a,b). These results indicated that PTX3 might contribute to cervical cancer progression.

### Knockdown of PTX3 inhibited cell proliferation in SiHa and HeLa cells

**Figure 2:**
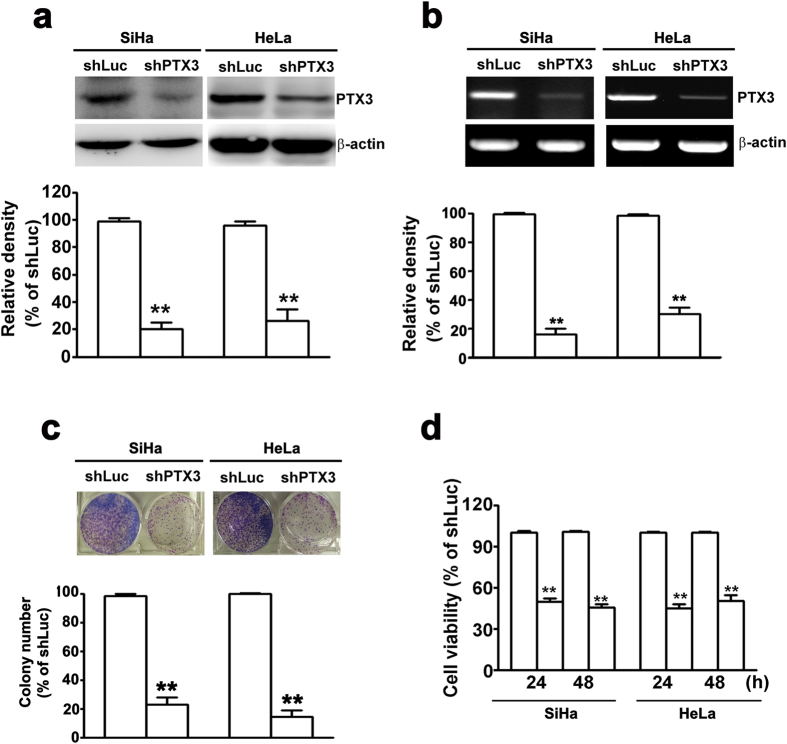
Knockdown of PTX3 inhibits cell growth in SiHa and HeLa cells. Knockdown of PTX3 expression in SiHa and HeLa cells was verified by (**a**) western blot and (**b**) RT-PCR assay. (**c**) Clonogenic assay was performed on SiHa and HeLa cells for 12 days. Upper panel shows representative images; and down panel shows quantification of colony numbers. (**d**) Cell proliferation rate for SiHa and HeLa cells at 24 and 48 h by MTT assay. The data represent the mean ± SE of three different experiments. **P < 0.01.

We found that higher expression level of PTX3 in HeLa and SiHa cells (Fig. 1b,c). Next, we inhibited the endogenous expression of PTX3 in HeLa and SiHa cells by shRNA assay to further investigate the biological function of PTX3 on human cervical cancer cells. Moreover, knockdown of PTX3 significantly inhibited the protein and mRNA expression of PTX3 in SiHa and HeLa cells by western blotting (Fig. 2a) and RT-PCR assay (Fig. 2b). The foci formation assay suggested larger and a higher quantity of colonies in the shPTX3 cells compared to the shLuc cells (Fig. 2c). The cell growth assay showed that cell growth rates in shPTX3 cells were significantly lower than those in the shLuc cells (Fig. 2d). Collectively, these results suggested that knockdown of PTX3 could inhibit cervical cancer cell proliferation.

### Knockdown of PTX3 induced cell cycle arrest at G2/M phase in SiHa and HeLa cells

**Figure 3:**
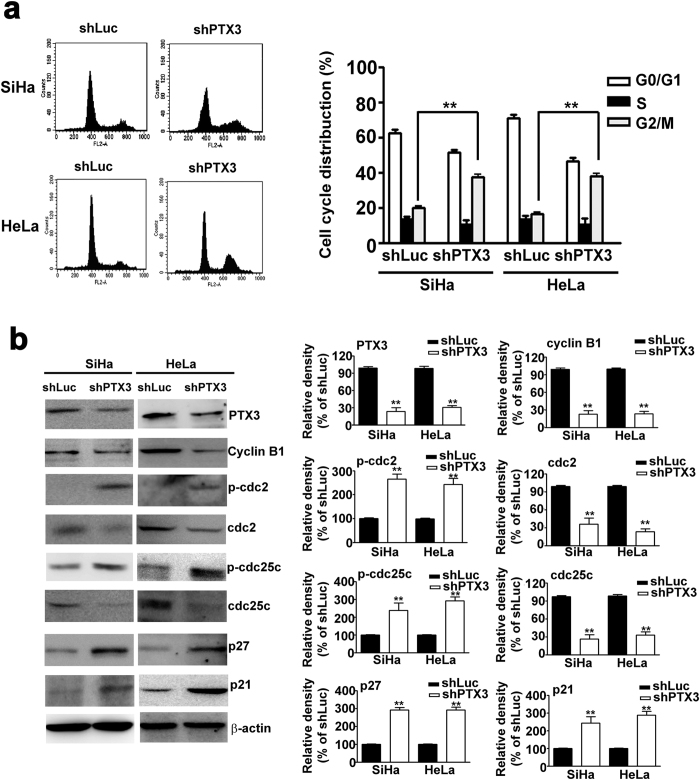
Knockdown of PTX3 induces cell cycle arrest G2/M phase of SiHa and HeLa cells. (**a**) Flow cytometry analysis of knockdown PTX3 in SiHa and HeLa cells. Right panel shows quantification of cell cycle distribution. (**b**) Western blotting analysis of PTX3, cyclin B1, p-cdc2, cdc2, p-cdc25c, cdc25c, p21, and p27 protein in knockdown PTX3 cells. β-actin served as the loading control. Right panel shows quantification of protein expression. The data represent the mean ± SE of three different experiments. **P < 0.01.

As shown in Fig. 3a, knockdown of PTX3 was associated with an increase of cells entering G2/M cell cycle phase (39.8%) in shPTX3-SiHa cells compared to shLuc-SiHa cells (20.5%). This increase was consistent with the percentage of cells entering G2/M phase (37.6%) in shPTX3-HeLa cells compared to shLuc-HeLa cells (15.4%). These results indicated that knockdown of PTX3 in SiHa and Hela cells could induce cell cycle arrest at the G2/M phase. Subsequently, we studied the effect of knockdown of PTX3 on the expression of G2/M related proteins in SiHa and Hela cells. Western blot analysis demonstrated that knockdown of PTX3 could downregulate the levels of cyclin B1, cdc2, and cdc25c, and upregulate the levels of p-cdc2, p-cdc25c, p21 and p27 (Fig. 3b). These results suggest that knockdown of PTX3 could induce cell arrest by modulating the G2/M phase cell-cycle related protein expression.

### Inhibition of PTX3 could inhibit the migration and invasion of SiHa and HeLa cells

**Figure 4:**
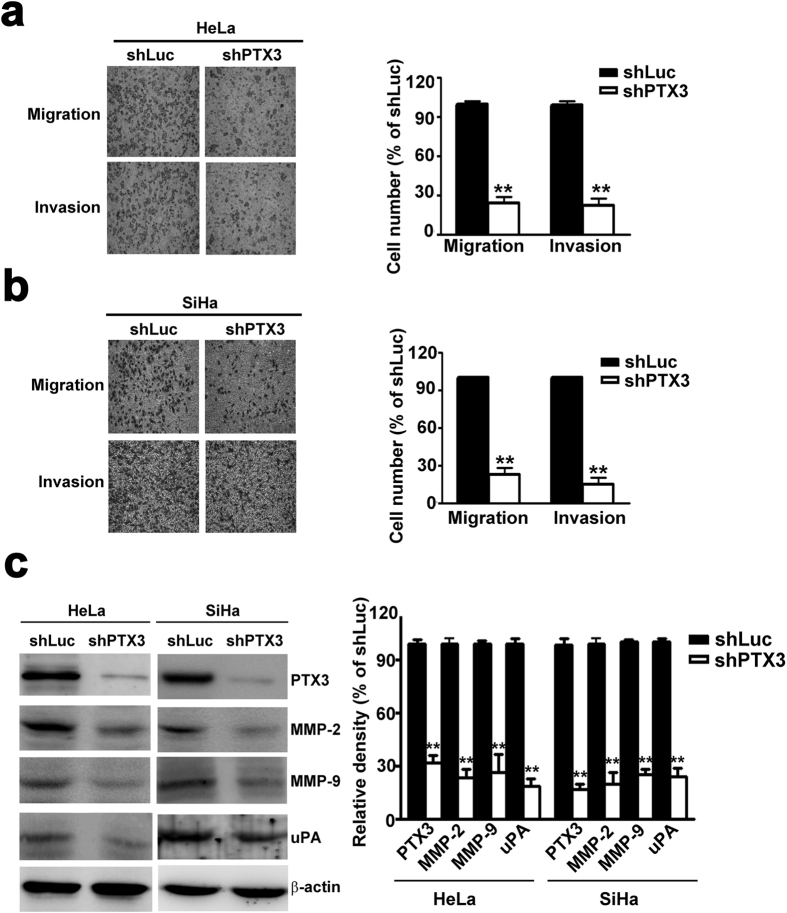
Knockdown of PTX3 inhibits cell migration and invasion in SiHa and HeLa cells. (**a**) Representative photographs of migratory and invasive cells on the membrane in cell migration and invasion assay. Average migratory cell numbers of shPTX3-SiHa cells were significantly lower than that of shLuc-SiHa cells. (**b**) Average migratory cell number of shPTX3-HeLa cells was significantly lower than that of shLuc-HeLa cells (original magnifications: ×40). (**c**) Western blotting analysis of PTX3, MMP-2, MMP-9, and uPA protein in knockdown of PTX3 cells. Right panel shows quantification of protein expression. The data represent the mean ± SE of three different experiments. **P < 0.01.

Quantification analysis indicated that migration and invasion was reduced by 74.1% and 79.2% in shPTX3-SiHa cells compared to shLuc-SiHa cells (Fig. 4a). Knockdown of PTX3 reduced cell migration and invasion by 73.4% and 75.2% in shPTX3-HeLa cells, respectively (Fig. 4b). Western blotting showed that knockdown of PTX3 in HeLa and SiHa cells significantly decreased the protein expression of MMP-2, MMP-9 and uPA (Fig. 4c). These results indicated that PTX3 plays an important role in cell migration and invasion.

### Overexpression of PTX3 induces the proliferation, migration and invasion activity of human cervical cancer cells

**Figure 5:**
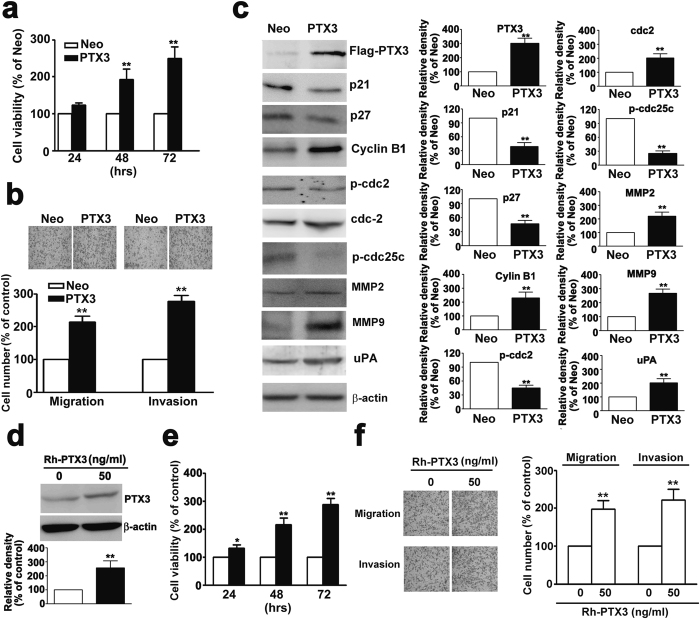
Overexpression of PTX3 induces cell proliferation, migration and invasion in human cervical HeLa cells. (**a**) HeLa cells were transfected with 3 μg of PTX3 plasmid and vector only (Neo) by lipofectamine agents. Cell viability was detected for 24, 48 and 72 h by MTT assay. (**b**) The migration and invasion of PTX3 and Neo cells were measured by *in vitro* migration and invasion assay. The non-migratory cells were removed and migrating cells were fixed and stained by crystal violet staining. The images were examined using a microscope (upper panel). (**c**) The expression levels of cell cycle related proteins (p21, p27, cyclin B, p-cdc2, cdc2, p-cdc25c, cdc25c) and invasive-related proteins (MMP2, MMP9, and uPA) in PTX3 over-expressing HeLa cells by western blotting (**d**) HeLa cell lines were treated with 50 ng/ml PTX3 for 48 h. Total lysates of cells were analyzed by western blotting with antibodies against PTX3 and b-actin protein (**e**) Cell viability were detected for 24, 48 and 72 h by MTT assay. (**f**) The migrates and invasive properties of HeLa cells were treated with 50 ng/ml PTX3 for 48 h, and examined using *in vitro* migration and invasion assay. Values represent the mean ± S.E. of three determinations.

Cell viability after transfection for 48 h was significantly increased in PTX3-overexpressing cells compared to Neo cells by MTT assay analysis (Fig. 5a). Western blotting showed an overexpressing PTX3 significantly decreased the expression of p21, p27, p-cdc2, and p-cdc25c and resulted in a significant increase in the expression of cdc2, cdc25c, and cyclin B1 (Fig. 5c). Overexpression of PTX3 significantly induced cell migration and invasion compared to Neo cells (Fig. 5b). The protein expression of MMP-2 MMP-9 and uPA also increased in PTX3-overexpressing cells compared to Neo cells (Fig. 5c).

Next, recombinant-PTX3 (Rh-PTX3) was applied to the human cervical HeLa cell line. Western blot analysis showed that cells treated with Rh-PTX3 had increased PTX3 expression (Fig. 5d). In Rh-PTX3-treated HeLa cells, the rate of cell growth was significantly higher than in control cells (Fig. 5e). The migration and invasion assay analysis showed that HeLa cell migration and invasion was strongly increased by Rh-PTX3 expression compared to control cells (Fig. 5f). To further confirm the oncogenicity of PTX3, we treated Rh-PTX3 in C33A cells, which exhibit relatively low expression of PTX3 among cervical cell lines. The MTT assay was showed a significantly higher proliferation rate than control cells in a time dependent (Supplementary Fig. 2a), and not induces cell apoptosis (Supplementary Fig. 2b). The *in vitro* migration and invasion assays were suggested that increased the migration and invasion potential of Rh-PTX3-treated C33A cells than control cells (Supplementary Fig. 2c). These results suggest that PTX3 overexpression may promote cell proliferation and invasion in human cervical cancer cells.

### PTX3 knockdown and overexpression on tumorigenesis and metastasis *in vivo*

**Figure 6:**
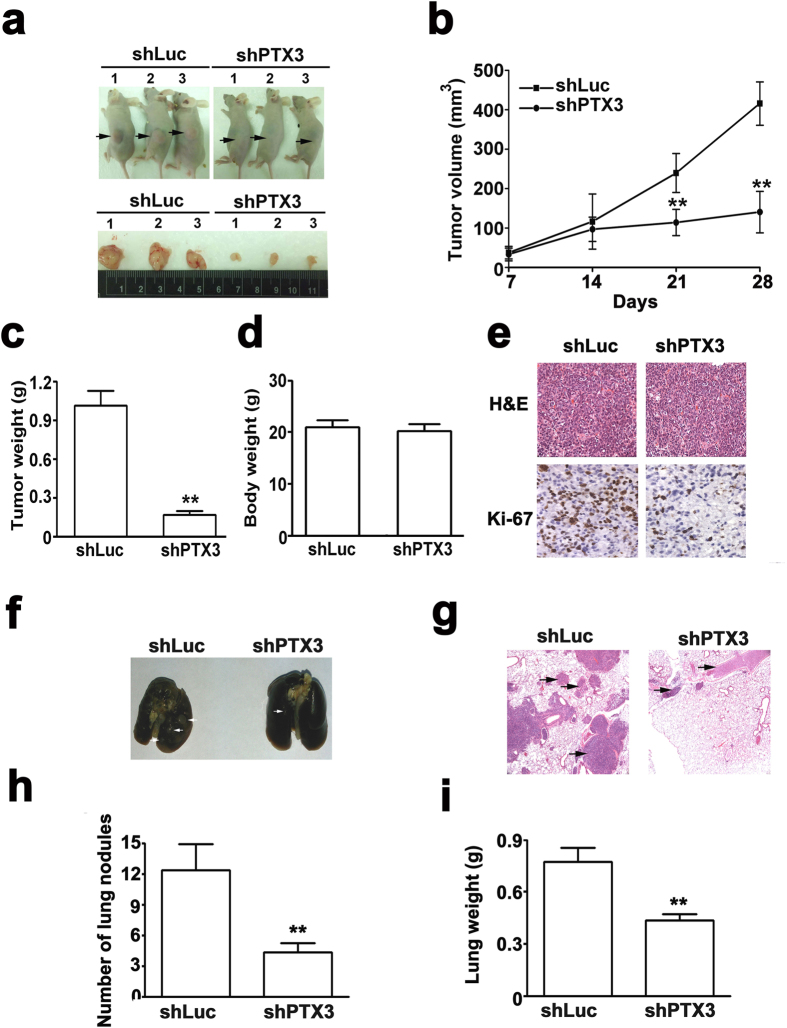
Knockdown of PTX3 suppressed tumorigenesis and metastasis *in vivo*. (**a**) For the xenograft, a total of 5 × 10^6^ cells were injected subcutaneously into the left flank, respectively. Photographs of tumors from mice in each group (n = 5/group) for 4-weeks. (**b**) Tumor growth curves were measured after injection, and tumor diameters were measured every 7 days. (**c**) Histograms of the mean tumor weights and (**d**) body weight of each group. Mean tumor weight and body weight for each group was calculated at 4 weeks after injection. (**e**) Representative images of H&E staining and Ki-67 expression of shLuc and shPTX3 group. (**f**) Photographs of lungs of the mice from each group were removed and (**g**) sectioned for evaluation lung metastasis after H&E staining. (**g**) H&E stained images of metastatic clusters in lungs from all groups with magnification of the selected areas. Representative images of H&E staining of tumor xenografts (magnifications: ×40). (**h**) There were lung metastatic colonies in the shPTX3 group significantly fewer than in the shLuc group. (**i**) Average lung weight of the mice from each group were removed and calculated. **P < 0.01.

**Figure 7:**
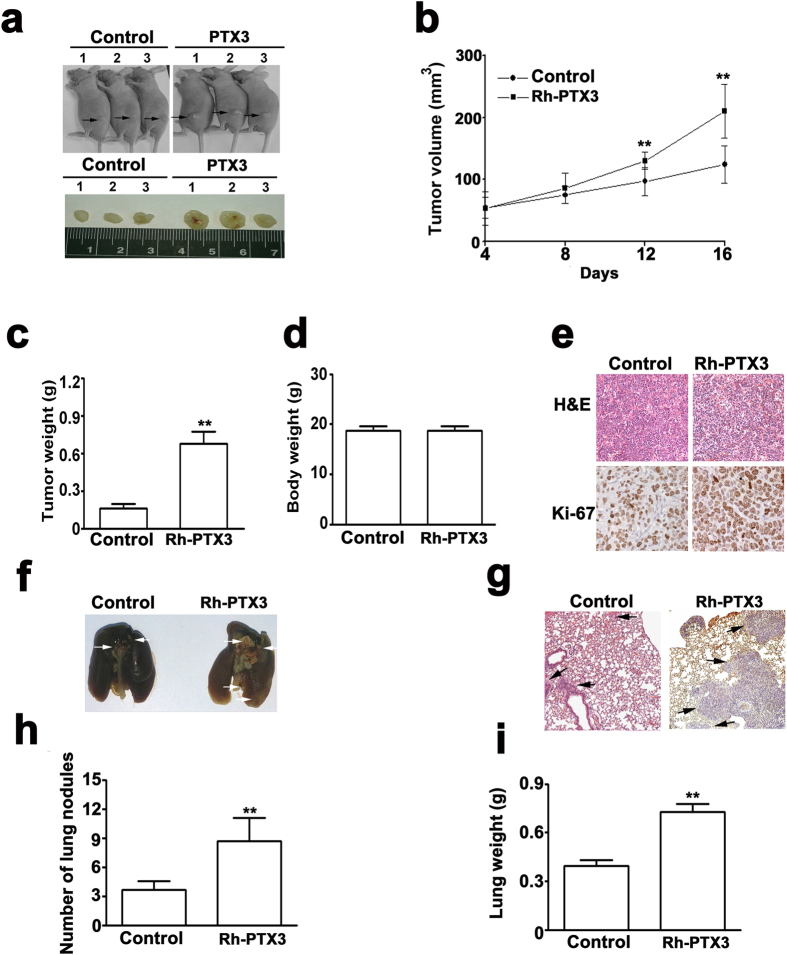
PTX3 promotes tumorigenesis and metastasis *in vivo*. (**a**) For the xenograft, a total of 5 × 10^6^ control (PBS) and Rh-PTX3 treated mices were injected subcutaneously into the left flank, respectively. Photographs of tumors from mice in each group (n = 5/group) for 16 days. (**b**) Tumor growth curves were measured after injection, and tumor diameters were measured every 5 days. **(c)** Mean tumor weight and **(d)** body weight for each group was calculated at 16 days after injection. (**e**) Representative images of H&E staining and Ki-67 expression of control and Rh-PTX3 group. (**f**) Photographs of lungs of the mice from each group were removed and (**g**) sectioned for evaluation lung metastasis after H&E staining. Representative images of H&E staining of tumor xenografts (magnifications: ×40). (**h**) There were lung metastatic colonies in the control group significantly fewer than in the Rh-PTX3 group. (**i**) Average lung weight of the mice from each group were removed and calculated. **P < 0.01.

The tumor growth of tumor xenografts incubated with shPTX3-SiHa cells significantly reduced tumor growth compared with shLuc-SiHa cells *in vivo*, (Fig. 6a,b). Additionally, the average weight of tumor xenografts incubated with shPTX3-SiHa cells was significantly less than that of the shLuc-SiHa cells (Fig. 6c). Ultimately, there was no difference in body weight between shLuc- and shPTX3-SiHa cells incubated mice (Fig. 6d). The expression of Ki-67 in the xenografts of shPTX3-SiHa cells was markedly lower than that observed in the xenografts of shLuc-SiHa cells by IHC assays (Fig. 6e). To assess the impact of PTX3 overexpression on the tumorigenesis of cervical cancer cells *in vivo.* The results of these experiments indicated that the tumor significantly larger in the xenografts of Rh-PTX3-SiHa cells than those in the xenografts of control cells at 16 days (Fig. 7a,b), and it significantly increased the tumor weight (Fig. 7c) and the expression of Ki-67 than those in the xenografts of control cells (Fig. 7e), whereas no was observed in different in body weight between the xenografts of control cells and Rh-PTX3-treated SiHa cells mice (Fig. 7d). These data thus validate the *in vitro* results that PTX3 contributes to increased tumorigenesis.

To examine the effect of the metastatic inhibiting ability of the knockdown of PTX3 on cervical cancer SiHa cells, two groups of 5 mice each were injected intravenously in the tail vein with shLuc- or shPTX3-SiHa cells, respectively. After 63 days, the mice were sacrificed, and the metastatic nodules at the lung surfaces were counted. As shown in Fig. 6f, the imaging of mice after injection showed that the incidence of lung metastasis was lower in mice injected with shPTX3-SiHa cells compared with shLuc-SiHa cells. H&E staining suggested that the nodules on the surfaces of mice lungs were metastatic tumors (Fig. 6g). Moreover, SiHa cells knocked down with PTX3 established statistically fewer lung metastatic colonies than cells in the shLuc group (P < 0.01, Fig. 6h). Additionally, the lung weight was decreased in shPTX3 group compared to the shLuc group (Fig. 6i). To demonstrate whether PTX3 overexpressing could increases metastasis *in vivo*. After 35 days, the mice were sacrificed, and the metastatic nodules at the lung surfaces were counted. We found that lung metastasis and colonies were significantly higher in RhPTX3-treated SiHa cells than those with the control SiHa cells (Fig. 7f,h). H&E staining showed that metastatic tumors of mice lungs in RhPTX3-treated SiHa cells, compared with control SiHa cells (Fig. 7g). Increase of lung weight was observed in Rh-PTX3 treated group (Fig. 7i). These results indicated that PTX3 plays a role in cervical cancer tumorigenesis and metastasis.

## Discussion

The human PTX3 gene, which encodes for a transcript of 1861 base pairs, has promoters involved in the response to proinflammatory cytokines^19^ and participates in tissue repair and remodeling^20^. PTX3 functions as a complement activator and facilitates pathogen recognition by phagocytes. It has been known that elevated serum PTX3 levels were a candidate biomarker for lung^8^, breast^21^, and prostate cancer^22^. PTX3 expression has been associated with tumor invasiveness, metastasis, angiogenesis, and as a prognostic factor^17^. By knocking down PTX3, the siRNA transfected cells displayed decreased PTX3 levels and suppressed the migratory ability of breast cancer cells^16^. These findings suggest the potential role of PTX3 in the development and progression of human cancers. In our study, we demonstrated that the overexpression of PTX3 in cervical cancer tissues was significantly associated with tumor grade and tumor differentiation. We further confirmed that PTX3 inhibited the growth and metastasis of cervical cancer cells *in vitro* and *in vivo*, suggesting the tumor promoter role of PTX3 in cervical cancer.

Recent evidence supports the oncosuppressive role of PTX3 in cancer progression. Bonavita *et al*. demonstrated that PTX3-deficient mice treated with 7,12-dimethylbenz [α] anthracene/terephthalic acid or 3-methylcholantrene had an increase in the formation of sarcomas and an increase in the incidence and multiplicity of papillomas and skin carcinomas, it correlation with cancer-related inflammation, such as CCL2 and C5a production. The main sources of PTX3 were permeating through leukocytes and endothelial cells, not tumor cells. Therefore, PTX3 acts as an extrinsic oncosuppressor in regulating tumor promoting inflammation^23^. Ronca *et al*. showed that PTX3 overexpressed-transgenic mice inhibited tumor growth, angiogenesis, and metastasis, and suggested that the implications in cancer therapy of the small-molecule chemical NSC12 is a PTX3- derived anti-FGF small molecule^24^. On the contrary, knockdown of PTX3 suppresses growth of lung adenocarcinoma A549 and LETPα-2 cells^11^. Although our results differ from previous research, we cannot rule out the possibility that PTX3 may have multiple functions on tumor cell growth^23^,^24^,^25^. Our results support the implication that the oncogenic role of PTX3 is related to its promoter action on proliferation, which sets the basis for the identification of a PTX3 promoter that is essential for cervical cancer progression.

During tumor metastasis, the infiltration of tumor cells to distant destinations relies on their attachment to blood vessels^26^,^27^. Upregulation of the expression and activity of MMPs and uPA have been shown to play a key role in human cancers with invasive and metastatic capability^28^,^29^. Previous studies have indicated that overexpression of MMP-9, MMP-2, and uPA correspond to the severity of cervical cancer, which correlate with tumor invasion, and is associated with parametrium invasion and lymph nodes metastasis in cervical cancer tissues^30^. Recent studies have demonstrated that exogenous PTX3 enhanced the migratory potential of macrophages to breast cancer cells and PTX3 silencing blocked macrophage mobility toward breast cancer cells^16^. Knockdown of PTX3 by lentiviral vector-mediated PTX3 shRNA suppressed tumor invasion by inhibiting expression of Akt, NF-κB, proliferating cell nuclear antigen, and MMP-9^11^. Other reports suggested that endothelial growth factor (EGF)-induced PTX3 production through the activation of the PI3K/Akt and NF-κB pathways increased MMP-9 and fibronectin expression in head and neck cancer cell metastasis. Of note, anti-PTX3 antibodies blocked EGF-induced expression of MMP-9^17^. On the contrary, PTX3 overexpression inhibited tumor angiogenesis and metastasis in heterotopic and orthotopic FGF-dependent transgenic mice models^24^. In this study, we demonstrated that the knockdown of PTX3 suppressed the metastatic ability of cervical cancer cells, which is consistent with the previous notion that PTX3 is a potential therapeutic target for head and neck cancer^17^. Further investigations are warranted to elucidate the molecular mechanism of PTX3 in the metastasis of cervical cancer cells.

To the best of our knowledge, this is the first study to demonstrate the clinical and biological functions of PTX3 in cervical cancer. Additionally, PTX3 may be a tumorigenic agent that modulates tumor growth promotion and metastasis of cervical cancer *in vitro* and *in vivo*. PTX3 may serve as a potential therapeutic target for the treatment of cervical cancer.

## Additional Information

**How to cite this article**: Ying, T.-H. *et al*. Knockdown of Pentraxin 3 suppresses tumorigenicity and metastasis of human cervical cancer cells. *Sci. Rep.* **6**, 29385; doi: 10.1038/srep29385 (2016).

## Supplementary Material

Supplementary Information

## Figures and Tables

**Table 1:** Correlation between PTX3 expression and clinicopathological characteristics of cervical cancer patients.

Characteristic	Number of patients (%)		*p* Value
	PTX3 staining		
	Negative	Positive	
Total number of patients	19 (31.6)	41 (68.3)	
Age (year)			
＜46	10 (31.3)	22(68.7)	0.580
≧46	9 (32.1)	19 (67.9)	
Tumor Grade			
I + II	15 (45.5)	18 (54.5)	<0.011
III	4 (14.8)	23 (85.2)	
Differentiated			
Well	2 (25)	6 (75)	＜0.019
Moderately	15 (46.9)	17 (53.1)	
Poorly	2 (10)	18 (90)	

## References

[b1] Waggoner S. E. Cervical cancer. Lancet. 361, 2217–2225 (2003). 12842378 10.1016/S0140-6736(03)13778-6

[b2] Vale C. . Reducing uncertainties about the effects of chemoradiotherapy for cervical cancer: a systematic review and meta-analysis of individual patient data from 18 randomized trials. J Clin Oncol. 26, 5802–5812 (2008). 19001332 10.1200/JCO.2008.16.4368 PMC2645100

[b3] Hanahan D. & Weinberg R. A. Hallmarks of cancer: the next generation. Cell. 144, 646–674 (2011). 21376230 10.1016/j.cell.2011.02.013

[b4] Deban L. . Pentraxins in innate immunity: lessons from PTX3. Cell Tissue Res. 343, 237–249 (2011). 20683616 10.1007/s00441-010-1018-0

[b5] Inforzato A. . PTX3 as a paradigm for the interaction of pentraxins with the complement system. Semin Immunol. 25, 79–85 (2013). 23747040 10.1016/j.smim.2013.05.002

[b6] Locatelli M. . The long pentraxin PTX3 as a correlate of cancer-related inflammation and prognosis of malignancy in gliomas. J Neuroimmunol. 260, 99–106 (2013). 23664694 10.1016/j.jneuroim.2013.04.009

[b7] Stallone G. . Pentraxin 3: a novel biomarker for predicting progression from prostatic inflammation to prostate cancer. Cancer Res. 74, 4230–4238 (2014). 24950910 10.1158/0008-5472.CAN-14-0369

[b8] Diamandis E. P., Goodglick L., Planque C. & Thornquist M. D. Pentraxin-3 is a novel biomarker of lung carcinoma. Clin Cancer Res. 17, 2395–2399 (2011). 21257721 10.1158/1078-0432.CCR-10-3024

[b9] Willeke F. . Overexpression of a member of the pentraxin family (PTX3) in human soft tissue liposarcoma. Eur J Cancer. 42, 2639–2646 (2006). 16959485 10.1016/j.ejca.2006.05.035

[b10] Kondo S. . Clinical impact of pentraxin family expression on prognosis of pancreatic carcinoma. Br J Cancer. 109, 739–746 (2013). 23828517 10.1038/bjc.2013.348 PMC3738116

[b11] Hu F. Q. . Knockdown of the inflammatory factor pentraxin-3 suppresses growth and invasion of lung adenocarcinoma through the AKT and NF-kappa B pathways. J Biol Regul Homeost Agents. 28, 649–657 (2014). 25620175

[b12] Webb C. P. & Vande-Woude G. F. Genes that regulate metastasis and angiogenesis. J Neurooncol. 50, 71–87 (2000). 11245283 10.1023/a:1006466605356

[b13] Deryugina E. I., Bourdon M. A., Reisfeld R. A. & Strongin A. Remodeling of collagen matrix by human tumor cells requires activation and cell surface association of matrix metalloproteinase-2. Cancer Res. 58, 3743–3750 (1998). 9721888

[b14] Choong P. F. & Nadesapillai A. P. Urokinase plasminogen activator system: a multifunctional role in tumor progression and metastasis. Clin Orthop Relat Res. 415 Suppl, S46–58 (2003). 14600592 10.1097/01.blo.0000093845.72468.bd

[b15] Liotta L. A. & Stetler-Stevenson W. G. Tumor invasion and metastasis: an imbalance of positive and negative regulation. Cancer Res. 51, 5054s–5059s (1991). 1884381

[b16] Choi B. . Elevated Pentraxin 3 in bone metastatic breast cancer is correlated with osteolytic function. Oncotarget. 5, 481–492 (2014). 24457902 10.18632/oncotarget.1664 PMC3964223

[b17] Chang W. C. . PTX3 gene activation in EGF-induced head and neck cancer cell metastasis. Oncotarget. 6, 7741–7757 (2015). 25797258 10.18632/oncotarget.3482 PMC4480713

[b18] Huang H. C. . Licochalcone A inhibits the migration and invasion of human lung cancer cells via inactivation of the Akt signaling pathway with downregulation of MMP-1/-3 expression. Tumour Biol. 35, 12139–12149 (2014). 25149157 10.1007/s13277-014-2519-3

[b19] Bottazzi B. . The long pentraxin PTX3 as a prototypic humoral pattern recognition receptor: interplay with cellular innate immunity. Immunol Rev. 227, 9–18 (2009). 19120471 10.1111/j.1600-065X.2008.00719.x

[b20] Deban L. . Pentraxins: multifunctional proteins at the interface of innate immunity and inflammation. Biofactors. 35, 138–145 (2009). 19449441 10.1002/biof.21

[b21] Margheri F. . Systemic sclerosis-endothelial cell antiangiogenic pentraxin 3 and matrix metalloprotease 12 control human breast cancer tumor vascularization and development in mice. Neoplasia. 11, 1106–1115 (2009). 19794969 10.1593/neo.09934 PMC2745676

[b22] Ravenna L. . Up-regulation of the inflammatory-reparative phenotype in human prostate carcinoma. Prostate. 69, 1245–1255 (2009). 19444819 10.1002/pros.20966

[b23] Bonavita E. . PTX3 is an extrinsic oncosuppressor regulating complement-dependent inflammation in cancer. Cell. 160, 700–714 (2015). 25679762 10.1016/j.cell.2015.01.004

[b24] Ronca R. . Long-Pentraxin 3 Derivative as a Small-Molecule FGF Trap for Cancer Therapy. Cancer Cell. 28, 225–239 (2015). 26267536 10.1016/j.ccell.2015.07.002

[b25] Bonavita E., Mantovani A. & Garlanda C. PTX3 acts as an extrinsic oncosuppressor. Oncotarget. 6, 32309–32310 (2015). 26457877 10.18632/oncotarget.4845 PMC4741692

[b26] Otero-Estevez O. . Serum matrix metalloproteinase-9 in colorectal cancer family-risk population screening. Sci Rep. 5, 13030 (2015). 26264519 10.1038/srep13030 PMC4532998

[b27] Mook O. R., Frederiks W. M. & Van Noorden C. J. The role of gelatinases in colorectal cancer progression and metastasis. Biochim Biophys Acta. 1705, 69–89 (2004). 15588763 10.1016/j.bbcan.2004.09.006

[b28] Roggel F. . Minimal residual disease in breast cancer and gynecological malignancies: phenotype and clinical relevance. Recent Results Cancer Res. 162, 89–100 (2003). 12790324 10.1007/978-3-642-59349-9_8

[b29] Wang P. H. . Clinical significance of matrix metalloproteinase-2 in cancer of uterine cervix: a semiquantitative study of immunoreactivities using tissue array. Gynecol Oncol. 108, 533–542 (2008). 18177928 10.1016/j.ygyno.2007.11.018

[b30] Daneri-Navarro A. . Urokinase-type plasminogen activator and plasminogen activator inhibitors (PAI-1 and PAI-2) in extracts of invasive cervical carcinoma and precursor lesions. Eur J Cancer. 34, 566–569 (1998). 9713310 10.1016/s0959-8049(97)10038-7

