# Supplementary material for: Knockdown of Pentraxin 3 suppresses tumorigenicity and metastasis of human cervical cancer cells
Source: Sci Rep. 2016 Jul 5;6:29385. doi: 10.1038/srep29385 (PMC4932528; doi:10.1038/srep29385)
Supplement: Supplementary Information [file srep29385-s1.pdf]

Knockdown of Pentraxin 3 suppresses tumorigenicity and metastasis of human cervical cancer cells

Tsung-Ho Ying, Chien-Hsing Lee, Hui-Ling Chiou, Shun-Fa Yang, Chu-Liang Lin, Chia-Hung Hung, Jen-Pi Tsai, Yi-Hsien Hsieh

Supplementary Figure 1

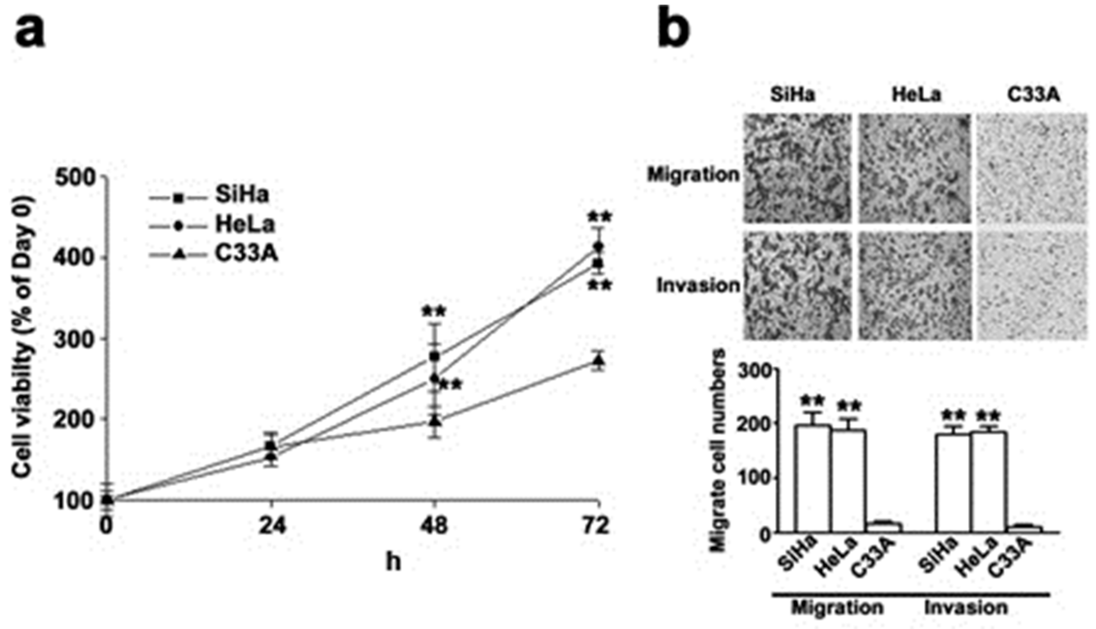

Supplementary Fig. 1 (a) The percentage of cell viability of SiHa, HeLa and C33A cells were estimated by MTT assay at 24, 48 and 72 h. **(b)** Representative photographs of migratory and invasive cells on the membrane in cell migration and invasion assay. Average migratory and invasive cell numbers of SiHa and HeLa cells were significantly higher than that of C33A cells. Results are shown as the mean ± SE of three independent experiments. ** $P < 0.01$.

Supplementary Figure 2

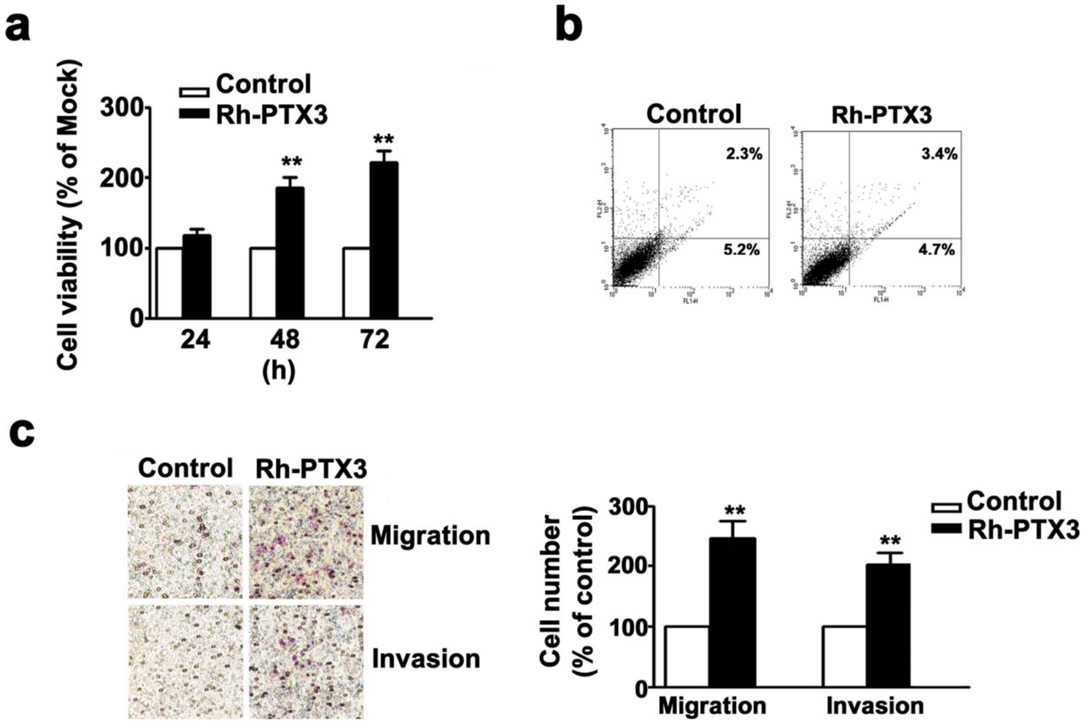

Supplementary Fig. 2. Overexpression of PTX3 promotes cell proliferation and invasion in C33A cells. **(a)** Cell viability in C33A cells treated with 100 ng/ml Rh-PTX3 were estimated by MTT assay at 24, 48 and 72 h. **(b)** Apoptosis induction in C33A cells treated with 100ng/ml Rh-PTX3 for 48 h was analyzed through Annexin-V/PI staining of by flow cytometry. **(c)** Migratory and invasive potential of C33A cells treated with 100 ng/ml Rh-PTX3 for 36 h. Results are shown as the mean ± SE of three independent experiments. ** $P < 0.01$, compared with control cells
